# Our Destroyer

**Published:** 1856-12

**Authors:** 


### OUR DESTROYER.

The New York *Herald*, which makes itself called for all over the country by its prompt and liberal publication of documents of general and scientific interest, in an article on the mortality of New York, states a fact which will surprise many who look at the mere surface of things, that for the single week ending November 1st, 1856, not less than seventy-two persons died of diseases of the brain and nervous system; the week preceding numbered seventy. The maladies of the mind, how terrible! How does anxiety and care, and wounded pride, and vain ambition eat out the hearts and drink up the life blood of the stirring thousands of a large city! Medicine cannot cure these nervous diseases; in the vain attempt to do so, many lives are sacrificed every year. Employment, *employment*—that is what they want, such as will compel the mind away, pleasurably, to steady physical activities.

